# Chemical Fingerprint and Metabolic Profile Analysis of Tianshu Tablets by Ultra-High Performance Liquid Chromatography/Quadrupole-Time of Flight Mass Spectrometry

**DOI:** 10.1155/2019/9158942

**Published:** 2019-10-23

**Authors:** Lin Chen, Renhao Chen, Hui Ouyang, Qi Wang, Zhifeng Li, Yulin Feng, Shilin Yang

**Affiliations:** ^1^College of Pharmacy, Jiangxi University of Traditional Chinese Medicine, Nanchang 330004, China; ^2^State Key Laboratory of Innovative Drug and Efficient Energy-Saving Pharmaceutical Equipment, Jiangxi University of Traditional Chinese Medicine, Nanchang 330006, China

## Abstract

In recent years, the chemical fingerprinting of traditional Chinese medicines and the metabolites in these compounds has been a hot topic. In the present study, the chemical fingerprint of Tianshu tablets (TST) and the metabolic characteristics of compounds in rats after intragastric administration were studied by ultra-high performance liquid chromatography coupled with quadrupole-time of flight mass spectrometry (UPLC/Q-TOF MS). In a preliminary study, 77 chemical components in TST were determined by comparison with retention times, accurate molecular mass, and characteristic fragment ions of the known compounds in the literature and some well-known compounds were analyzed in detail, and the fragmentation pathways for parishins B, gastrodin A, and cnidilide or neocnilide were specifically analyzed. After intragastric administration of TST (4 g/kg) to rats, a total of 61 compounds were detected in plasma samples, including 7 prototypes and 54 metabolites. After further analysis, it was found that these metabolites were subjected to glucuronidation, sulfation, methylation, hydroxylation, dehydrogenation, or mixed metabolic processes. Hydroxylation and glucuronidation were finally confirmed as the main metabolic pathways. This is the first research on the chemical fingerprint and metabolites of TST, which lays a foundation for further investigation of TST.

## 1. Introduction

In recent years, traditional Chinese medicine (TCM) has attracted increasing attention worldwide by virtue of its applications. *Da Chuanxiong Formula* (DCXF) is a well-known and extensively used traditional Chinese medicine (TCM) decoction for the treatment of migraine caused by blood stasis and wind-heat syndrome. It is composed of two herbs, namely, Chuanxiong (*Chuanxiong rhizoma*) and Tianma (*Gastrodiae rhizoma*), with a crude weight ratio of 4 : 1. Tianshu tablets (TST) are a representative DCXF preparation that is widely used in clinics for treating the blood stasis type of headache and migraine [1–3].

Phytochemical and pharmacological investigations of DXCF have shown that phenols, organic acids, phthalides, and nitrogen-containing compounds are the major active ingredients [4]. At present, several qualitative studies on the main components of DCXF have been performed [5–8]. One study used LC-Q-TOF/MS to identify 17 different components in a 50% ethanol extract of DCXF [5]. In one study, three compounds of Chuanxiong and eight components of Tianma were identified by HPLC-DAD-MS^n^ [6]. Two continuous studies showed that 10 different compounds were detected in rat plasma after intragastric administration of DCXF active components, including 6 compounds from Chuanxiong and 4 compounds from Tianma [7, 8]. These four studies were based on samples of a 50% ethanol extract from a 4 : 1 mixture of the two herbs or active ingredients from a single crude herb. In one study, 38 components were identified or preliminarily identified from a Tianshu capsule by means of HPLC, LC-DAD-MSN, and LC-DAD-ESI IT-TOF/MS analysis, although Tianshu tablets and Tianshu capsules are two different dosage forms [9]. This research is still very important because of its different applicability. This study enriches our understanding of the components of DCXF and studies the metabolites of TST for the first time.

In our study, 77 chemical components of TST were preliminarily determined by a comparison with retention time, accurate molecular mass, and characteristic fragment ions of known compounds in the literature. Furthermore, UPLC/Q-TOF MS was used to analyze the plasma of rats after oral administration of TST. A total of 61 compounds were identified or preliminarily identified, including 7 prototypes and 54 metabolites.

## 2. Experimental

### 2.1. Chemicals and Materials

Some reference standards (pyroglutamic acid, 5-(hydroxymethyl)furoic acid, and parishin B) for *Gastrodia rhizoma* were isolated and purified in our laboratory, and other standards (uridine, gastrodin A, and neocnilide) were purchased from the National Institutes for Food and Drug Control (Beijing, China). Purities of the standards were above 98% by HPLC analysis. HPLC-grade acetonitrile, methanol, and formic acid were purchased from Fisher Scientific (MA, USA). Deionized water was prepared by a Milli-Q Water purification system (Millipore, MA, USA). High-purity nitrogen (99.99%) and helium (99.999%) were purchased from Gas Supplies Center of Peking University Health Science Center (Beijing, China).

*Gastrodia rhizoma* and *Chuanxiong rhizoma* were purchased from Tian Heng pharmacy (Beijing, China). All herbal materials were authenticated by Professor Bei Wu (Nanchang Institute for Food and Drug Control). TianShu tablets were prepared according to Chinese Pharmacopoeia 2015 Edition [1].

### 2.2. Animals and Drug

Sprague–Dawley rats (male, 12–14 weeks; 200–240 g) were provided by Hunan SJA Laboratory Animal Co., Ltd. Protocols for all animal experiments were approved. Animals were kept in a controlled environment for 3 days and fasted for 12 h before experiments. TST were dissolved in a 9 g/L NaCl solution (NS) (250 mg/ml) and administered by oral gavage at a dose of 1000 mg/kg (equivalent to 4 g of crude drug per kg) body weight.

### 2.3. Sample Collection and Pretreatment

After oral administration of TST, blood samples were collected at 30, 60, and 120 min (*n* = 5) in an Eppendorf tube with heparin sodium and then centrifuged (16000 rpm) at 4°C for 10 min. The supernatant was then separated, and all samples were stored at −80°C immediately until analysis. The protocol for sample preparation is described below: 1 mL plasma was mixed with 5 mL methanol, vortexed for 5 min, and centrifuged at 16000 rpm at 4°C for 20 min. The supernatant was dried with nitrogen gas at 4°C, and 400 *μ*l of 50% methanol was added to reconstitute the residue, which was then centrifuged at 16000 rpm for 10 min at 4°C. The supernatant was transferred to a vial, and 10 *μ*l was injected for LC-MS analysis. All samples were filtered through a membrane (0.22 *μ*m pore size). At the same time, in order to eliminate the influence of matrix, blank plasma was added to participate in the analysis.

### 2.4. UPLC/QTOF-MS Conditions

UPLC/QTOF-MS analysis was performed on a Shimadzu LC-30 AD system (Kyoto, Japan) coupled with an AB SCIEX Triple-TOF 5600 mass spectrometer (Foster City, CA, USA). All samples were separated on an Acquity UPLC C18 column (100 mm × 2.1 mm, 1.7 *μ*m, Waters, USA) with a flow rate of 0.3 mL/min at 40°C. The mobile phase consisted of aqueous 0.1% formic acid (A) and 0.1% formic acid in acetonitrile (B). The gradient elution program for TST was set at 0–3.0 min, 5–8% B; 3.0–10.0 min, 8–15% B; 10.0–18 min, 15–20% B; 18–22 min, 20–35% B; 22–37 min, 35–45% B; 37–43 min, 45–95% B; and 43–48 min, 95–95% B. The gradient elution program for the plasma samples was set at 0–2.0 min, 5% B; 2.0–25.0 min, 5–95% B; and 25.0–30.0 min, 95–95%. The equilibration time was 5 min. The conditions for the ion source were as follows: compounds in TST were measured using the total ion chromatograms in negative and positive ion ESI-MS mode in the mass range *m*/*z* 50–1250, but plasma samples were analyzed only in positive ion ESI-MS mode. The other operating parameters were optimized as follows: source temperature, 500°C; ion spray voltage, 4500 V; gas 1, 50 psi; gas 2, 50 psi; curtain gas, 45 psi; decluttering potential, 100 V; and collision energy was set to 40 (15) eV.

### 2.5. Data Process

TST compounds from the extracts and metabolites data were acquired by full scan, which rely on dynamic background subtraction (DBS) and multiple mass defect filtering (MMDF) and includes some compounds with very low concentrations (MDF window was set to ±50 mDa around the mass defects of the templates and over a mass range of ±50 Da around the filter template masses).

Analysis of data on TST compounds in extracts and metabolites was performed using a variety of data mining tools, including extract ion chromatograms (XIC) of PeakView®1.6 (AB SCIEX, CA, USA), MMDF, and NLF&DPLs of Metabolitepilot™ 1.5 (AB SCIEX, Foster City, CA, USA). All compounds were analyzed after removal of the matrix effects.

## 3. Results and Discussion

### 3.1. Optimization of LC/MS Conditions

In order to obtain the best analytical data, our analysis builds upon another recent study [10]. The separation conditions, supplements, and chromatographic columns were optimized at the beginning of the experiment. Firstly, in order to obtain sharp peaks and reduce the pressure on the UPLC column, methanol was used as the mobile phase instead of acetonitrile. At the same time, 0.1% formic acid was added to improve peak shape and ionization of the analytes. The gradient was improved, and it was shown that the compounds in TST could be separated within 48 minutes, while plasma samples could be separated within 30 minutes (the specific methods can be found in Section 2.4). In addition, in order to obtain the most abundant mass spectrometry information, the collision energy was optimized. The results showed that when the collision energy rose to 40 eV, the main fragments were seen, but when the energy reached 55 eV, the second order fragments were too fragmented to be easily analyzed. Therefore, a collision energy of 40 eV was selected. As for UPLC/Q-TOF MS, mass spectra were recorded in both positive and negative detection modes.

### 3.2. UPLC/Q-TOF MS Analysis of TST Extracts

**Figure 1:**
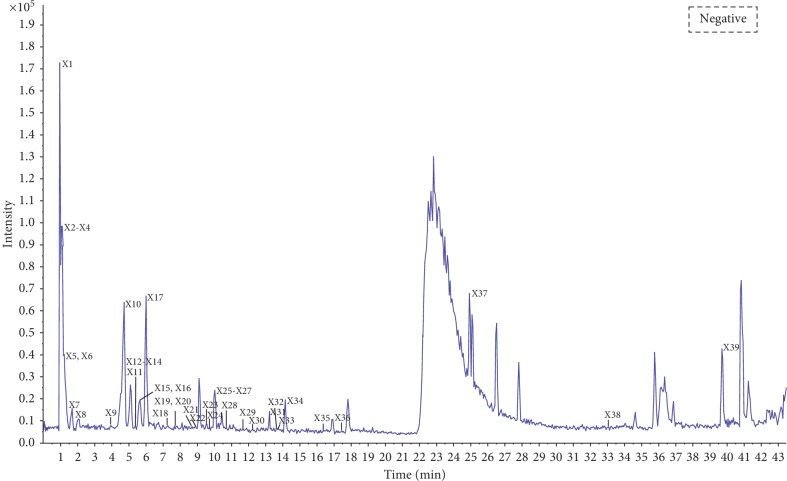
MS total ion chromatogram (TIC) of TST fraction by UPLC/Q-TOF MS in negative ion mode.

**Figure 2:**
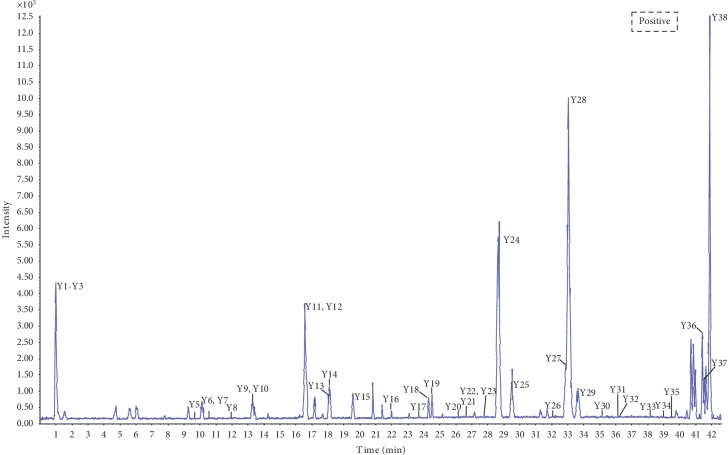
MS total ion chromatogram (TIC) of TST fraction by UPLC/Q-TOF MS in positive ion mode.

To characterize the chemical constituents of TST, a fast, efficient, and reliable UPLC/Q-TOF MS method was established. By virtue of the high resolution and speed of UPLC and the accurate mass measurement of the TOF MS, a total of 77 compounds were identified. The mass spectra of these components were examined in negative ion mode and positive ion mode. The total ion chromatogram (TIC) of TST in positive and negative ion modes are shown in Figures 1 and 2. Details of the identified components are summarized in Table 1.

**Figure 3:**
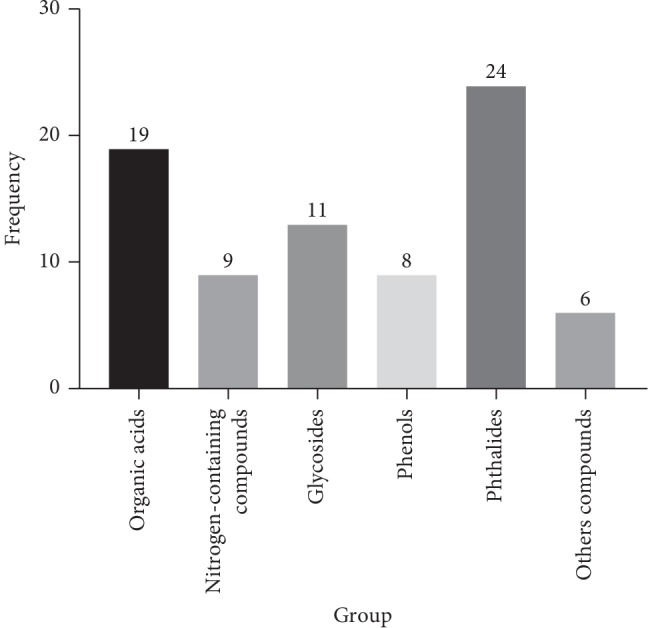
Information about classification of compounds in TST.

Through analysis, it was found that the 77 compounds contained 19 organic acids, 9 nitrogen-containing compounds, 11 glucosides, 8 phenols, 24 phthalides, and 6 other compounds. The numbering information of these compounds is shown in Figure 3.

#### 3.2.1. Chemical Fingerprint of TST in Negative Ion Modes

According to the literature, the main components of Tianma are phenols and organic acids [9]. However, there are also glycosides in the components of Tianma [12]. Many characteristic components of Tianma were analyzed and identified in the negative ion mode. Because the structures of organic acids and phenols are relatively simple, the characteristic glycoside compounds **X18** and **X23** were identified here and the chromatographic and spectral data for compounds **X18** and **X23** were preliminarily characterized by referring to the literature and reference materials.

**Figure 4:**
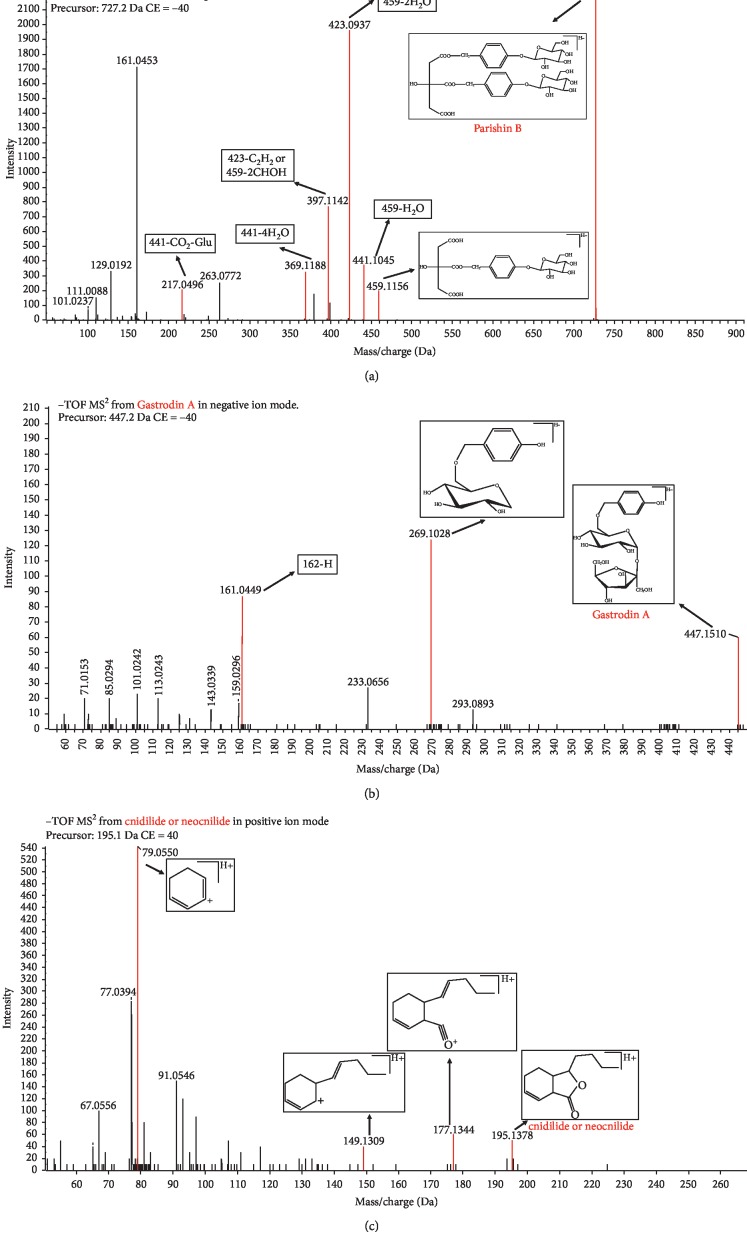
MS/MS spectra and the proposed fragmentation pathways. (a) Parishin B in negative ion mode. (b) Gastrodin A in negative ion mode. (c) Cnidilide or neocnilide in positive ion mode.

Peak **X18** gave an [M−H]^−^ ion at *m*/*z* 447.1508. Peak **X18** produced MS^2^ base peaks at *m*/*z* 269.1028 and 161.0449 corresponding to [M-H-179 Da]^−^. This suggests that Peak **X18** may contain a glucose group and a fructose group. Therefore, we deduced that the molecular structure likely contains sucrose. According to literature reports [12] and reference standards, we identified Peak **X18** as gastrodin A. Peak **X23** gave a [M-H]^−^ ion at *m*/*z* 727.2091 and had characteristic fragment ions at *m*/*z* 459.1156, 441.1045, 423.0937, 397.1142, 369.1188, and 217.0496. Based on previous studies [12] and a reference standard, Peak **X23** was identified as parishin B. The characteristic fragmentation patterns of gastrodin A and parishin B are described in Figures 4(a) and 4(b).

#### 3.2.2. Chemical Fingerprint of TST in Positive Ion Modes

The analysis of the positive ion mode results showed that the characteristic components of Chuanxiong, including phthalides, were present. Here, compound Y27 was selected for analysis, and the chromatographic and spectral data of this compound were analyzed by comparison with the literature and reference materials. The cleavage pathway of phenyl peptides in Chuanxiong was also analyzed.

Peak **Y27** gave a [M+H]^+^ ion at *m*/*z* 195.1378 and fragment ions at *m*/*z* 177.1344, 149.1309, and 107.0550. According to previous literature reports [9] and a reference standard, we identified peak **Y27** as cnidilide or neocnilide. The characteristic fragmentation pattern of **Y27** is shown in Figure 4(c)

According to our analysis, the main components of Tianma in negative ion mode were organic acids, phenols, and glycosides, with mainly phthalides detected in positive ion mode. The specific pyrolysis fragments were similar to the standards.

### 3.3. Detection and Identification of the Metabolites of TST in Rat Plasma

**Figure 5:**
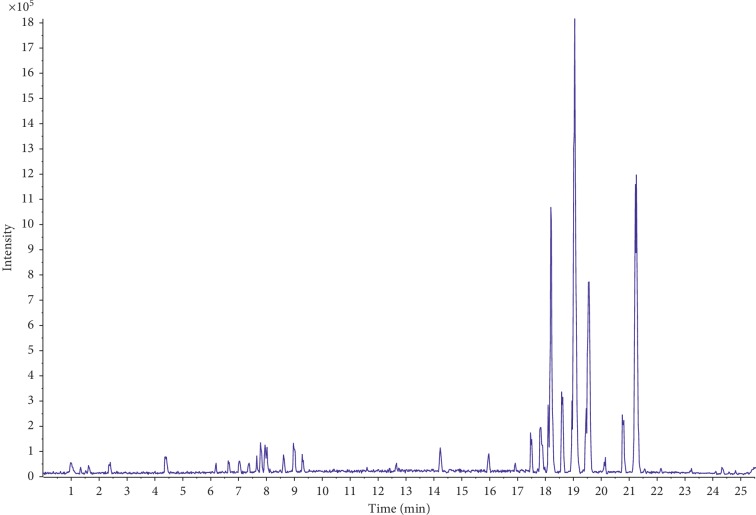
MS total ion chromatogram (TIC) of metabolites by UPLC/Q-TOF MS.

**Figure 6:**
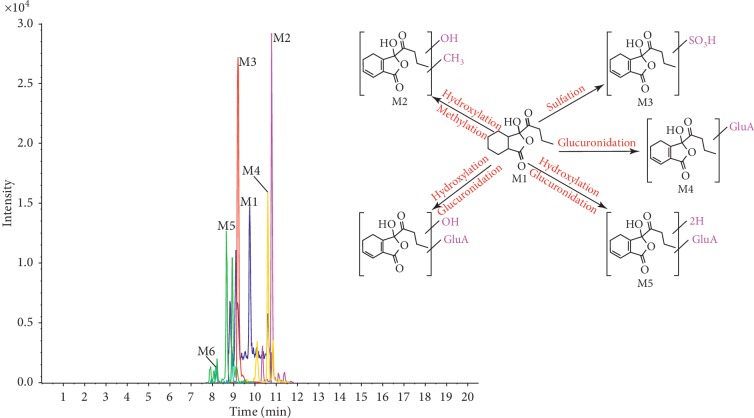
Metabolites of M1 and extract ion chromatogram (EIC).

**Figure 7:**
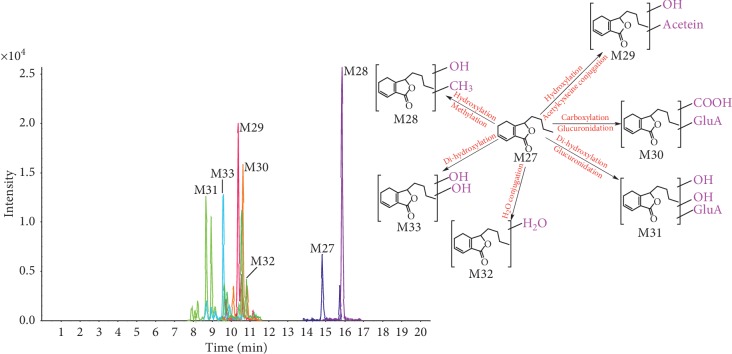
Metabolites of M27 and extract ion chromatogram (EIC).

**Figure 8:**
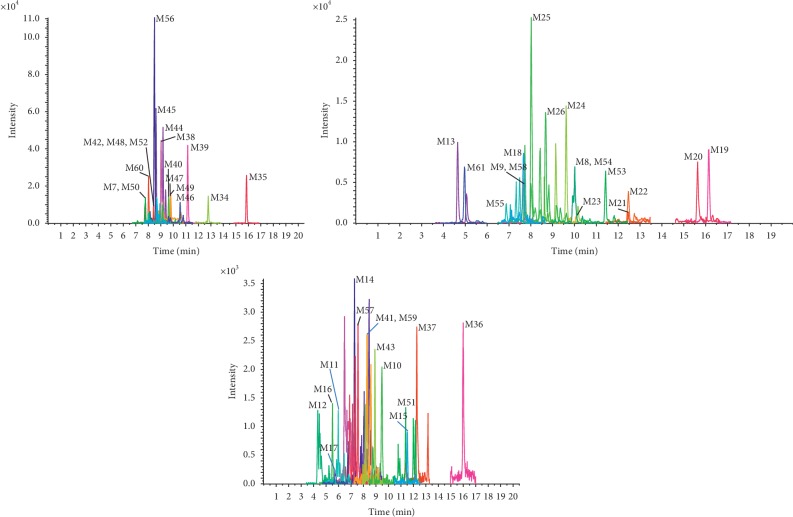
The extract ion chromatograms (EICs) of other metabolites detected.

In order to identify as many potential pharmacologically active compounds as possible in TST, metabolic profiling of TST in rat plasma was performed. Compounds absorbed in vivo can be further metabolized by a variety of enzymes through oxidation, hydrolyzation, methylation, glucuronidation, and sulfation. Only peaks that were detected in the dosed plasma samples but not in blank samples were considered as probable metabolites. The mass spectra of the metabolites were examined in positive ion mode. These were further analyzed by using Peakview 1.2 to identify expected and unexpected metabolites from different metabolic pathways, and their structures were identified by tandem MS. We selected senkyunolide D or 4,7-dihydroxy-3-butylphthalide and senkyunolide A as examples of the structural identification process. The metabolites of these compounds and others are summarized in Table 2, and their TIC and extract ion chromatogram (EICs) are shown in Figures 5–8.

Metabolite **M1**, which eluted at 9.75 min, formed a molecular ion of [M+H]^+^ at *m*/*z* 223.0963 corresponding to C_12_H_14_O_4_. **M1** was found to have major fragment ions in common with senkyunolide D, so **M1** is most likely senkyunolide D or 4,7-dihydroxy-3-butylphthalide. Metabolite **M2**, which eluted at 10.78 min, formed a molecular ion of [M+H]^+^ at *m*/*z* 253.1071 corresponding to C_13_H_16_O_5_. The characteristic production of *m*/*z* 235.0963 and 221.0839 was generated by loss of 18 Da and 18 + 14 Da, which implied loss of a H_2_O group and methyl group. Other product ions were identical to that of **M1**. Therefore, **M2** may be a metabolite of senkyunolide D after hydroxyl and methyl conjugation. Metabolite **M3**, which eluted at 9.2 min, formed a molecular ion of [M+H]^+^ at *m*/*z* 303.0535 corresponding to C_12_H_14_SO_7_. Its major fragment ions at *m*/*z* 285.0430 and 205.0858 were generated by the loss of 18 Da and 18 + 80 Da, which implied a H_2_O group and sulfate group. Other product ions were identical to that of **M1**. Therefore, **M3** may be a metabolite of senkyunolide D following sulfation. Metabolite **M4**, which eluted at 10.6 min, formed a molecular ion of [M+H]^+^ at *m*/*z* 399.1285 corresponding to C_18_H_22_O_10_. Its major fragment ion (*m*/*z* 223.0969) was generated by a loss of 176 Da, which implied a glucuronide group. Other product ions were identical to that of **M1**. Therefore, **M4** might be a metabolite of senkyunolide D after glucuronidation. Metabolites **M5** and **M6** appear to correspond to **M4** plus 2 Da or 16 Da, respectively. The product ions *m*/*z* 225.4427 and *m*/*z* 227.0584 have both lost 176 Da. Therefore, **M5** might be a metabolite of senkyunolide D after hydrogenation and glucuronidation, while **M6** might be a metabolite of senkyunolide D after hydroxylation and glucuronidation (metabolites of **M1** and extract ion chromatograms (EICs) are shown in Figure 6).

Metabolite **M27**, which eluted at 14.81 min, formed a molecular ion of [M+H]^+^ at *m*/*z* 193.1222 corresponding to C_12_H_16_O_2_. A major fragment ion was shared with senkyunolide A, suggesting that **M27** is prototype of senkyunolide A. Metabolite **M28**, which eluted at 14.81 min, formed a molecular ion of [M+H]^+^ at *m*/*z* 223.1328 corresponding to C_13_H_18_O_3_. Its major fragment ions *m*/*z* 205.1224 and 191.1060 were generated by loss of 18 Da and 18 + 14 Da, which implied loss of a H_2_O group and methyl group. Other product ions were identical to that of **M27**. Based on the possible metabolic reactions, **M28** might be a metabolite of senkyunolide A after hydroxylation and methylation. Metabolite **M29**, which eluted at 10.35 min, formed a molecular ion of [M+H]^+^ at *m*/*z* 372.1475 corresponding to C_17_H_25_O_6_NS. Its major fragment ions *m*/*z* 209.1269 and 191.1074 were generated by loss of 163 Da and 163 + 18 Da, which implied loss of an acetylcysteine group and H_2_O group. Other product ions were identical to that of **M27**. Therefore, **M29** may be a metabolite of senkyunolide A after hydroxyl and acetylcysteine conjugation. Metabolite **M30**, which eluted at 10.60 min, formed a molecular ion of [M+H]^+^ at *m*/*z* 399.1285 corresponding to C_18_H_22_O_10_. Its major fragment ions *m*/*z* 223.0969, 205.0847, and 177.0883 were generated by the loss of 176 Da, 176 + 18 Da, and 176 + 18 + 28 Da, which implied loss of a glucuronide group, H_2_O group, and CO group. Other product ions were identical to that of **M27**. Therefore, **M30** may be a metabolite of senkyunolide A following carboxylation and glucuronidation. Metabolite **M31**, which eluted at 8.67 min, formed a molecular ion of [M+H]^+^ at *m*/*z* 401.1444 corresponding to C_18_H_24_O_10_. Its major fragment ions *m*/*z* 225.1127, 207.1017, and 189.0924 were generated by loss of 176 Da, 176 + 18 Da, and 176 + 18 + 18 Da, which implied loss of a glucuronide group and two H_2_O groups. Other product ions were identical to that of **M27**. Therefore, **M31** might be a metabolite of senkyunolide A after 2 hydroxylation events and glucuronidation. Metabolite **M32**, which eluted at 10.54 min, formed a molecular ion of [M+H]^+^ at *m*/*z* 211.1327 corresponding to C_12_H_18_O_3_. Its major fragment ions were *m*/*z* 193.1225 and 175.1096. An *m*/*z* of 193.1225 (loss of 18 Da) corresponds to senkyunolide A, suggesting that **M32** might be a metabolite of senkyunolide A after H_2_O conjugation. Metabolite **M33**, which eluted at 9.56 min, formed a molecular ion of [M+H]^+^ at *m*/*z* 225.1119 corresponding to C_12_H_16_O_4_. Its major fragment ions *m*/*z* 207.1013 and 189.0914 were generated by loss of 18 Da and 18 + 18 Da, which implied loss of one or two H_2_O groups. Other product ions were identical to that of **M27**. Therefore, **M33** might be a metabolite of senkyunolide A after 2 hydroxylation events (metabolites of **M27** and extracted ion chromatograms (EICs) are shown in Figure 7).

Sixty-one metabolites were identified in rat plasma. Through the analysis of these 61 metabolites, it was found that hydroxylation and glucuronidation were the main metabolic ways following oral administration of TST. From the identified metabolites, it can be speculated that after absorption of TST by human blood, most of the compounds undergo hydroxylation and glucuronidation, which allow TST to play a positive role in the treatment of migraine and blood stasis headaches. This provides a basis for follow-up research on the medical uses of TST. At the same time, from the information obtained on the metabolites, it can be seen that the main metabolites in positive ion mode of TST are concentrated as chuanxiong lactones, but there are no effective metabolites from Tianma. It is possible that Tianma metabolites are mainly present in the negative ion mode of plasma or in feces, urine, and bile, which requires further study.

## 4. Conclusion

In this study, UPLC/Q-TOF MS was used to comprehensively determine the chemical fingerprint and metabolic profile of TST after intragastric administration. In the analysis of the chemical constituents of TST, 77 compounds were identified, including 39 compounds identified in negative ion mode and 38 compounds identified in positive ion mode. In order to elucidate the mass spectrometric pyrolysis law of the main compounds in TST, gastrodin A, parishin B, and cnidilide or neocnilide were specifically analyzed, and the results were completely consistent with the results in reference standards and the reported literature. And 61 metabolites of TST in rat plasma were detected, which were mainly metabolites of 7 compounds. Two prototypes (senkyunolide D or 4,7-dihydroxy-3-butylphthalide and senkyunolide A) and their metabolites were analyzed in detail, which showed hydroxylation and glucuronidation were the main metabolic pathways following oral administration. This study expanded our understanding of the chemical constituents of TST, studied its metabolic spectrum for the first time, and clarified its main metabolic pathway in plasma, which will lay the foundation for follow-up studies of the pharmacological mechanism of TST.

## Figures and Tables

**Table 1:** The chemical constituents detected from TST.

Peak	RT (min)	Formula	Error (ppm)	[M−H]^−^ (*m*/*z*)		Intensity	Product ions	Identification	Structure class	Ref.
				Calculated	Measure					
X1^a^	0.98	C_7_H_12_O_6_	−1.2	191.0561	191.0559	86129	154.9973, 127.0393, 111.0091, 93.0355, 85.0302, 73.0302	Quinic acid	Organic acids	[10]
X2	1.07	C_6_H_8_O_7_	0.9	191.0197	191.0199	421357	111.0096, 87.0103	Citric acid	Organic acids	[10]
X3^b^	1.07	C_9_H_12_N_2_O_6_	−1	243.0623	243.0620	5161	nd	Uridine	Nitrogen-containing compounds	
X4^b^	1.08	C_5_H_7_NO_3_	3	128.0353	128.0357	24352	nd	Pyroglutamic acid	Organic acids	
X5^b^	1.36	C_6_H_6_O_4_	1.1	141.0193	141.0195	3248	nd	5-(Hydroxymethyl)furoic acid	Organic acids	[11]
X6	1.36	C_10_H_13_N_5_O_5_	2.4	282.0844	282.0851	1153	150.0412, 133.0158, 108.0286	Guanosine	Nitrogen-containing compounds	
X7	1.63	C_13_H_18_O_7_	−1.1	285.0980	285.0977	1505	223.0248, 123.0432, 105.0348	Gastrodin	Glycosides	[12]
X8	2.03	C_7_H_6_O_4_	0.7	153.0193	153.0195	16114	109.0300, 91.0193, 65.0057	Protocatechuic acid	Organic acids	[13]
X9	4.02	C_7_H_6_O_3_	2.5	137.0244	137.0248	31464	119.0140, 108.0215, 93.0350, 81.0366, 65.0414	3,4-Dihydroxy benzaldehyde	Phenols	[14]
X10	4.70	C_19_H_24_O_13_	1.7	459.1144	459.1152	283440	423.0939, 397.1138, 173.0088, 61.0444, 129.0184, 111.0088	Parishin E or G	Glycosides	[12]
X11	5.09	C_16_H_18_O_9_	0.8	353.0878	353.0881	109164	191.0559, 173.0444, 161.0236, 134.0387, 111.0458, 93.0350, 85.0301	4-Caffeoylquinic acid	Organic acids	[15]
X12	5.13	C_17_H_23_N_3_O_7_S	0.5	412.1184	412.1186	11210	306.0753, 272.0898, 179.0459, 160.0077, 143.0461, 128.0348, 99.0559	*S*-(4-Hydroxybenzyl)-glutathione	Nitrogen-containing compounds	[12]
X13	5.24	C_23_H_33_N_3_O_12_S	2.3	574.1712	574.1726	7677	484.1388, 378.0974, 306.0756, 272.0874, 210.0864, 143.0464	*S*-(4-Hydroxybenzyl)-glutathione glucoside	Nitrogen-containing compounds	
X14	5.34	C_9_H_10_O_4_	−0.2	181.0506	181.0506	2573	136.9210, 121.0296, 109.0279	4-(2-Hydroxyethoxy)benzoic acid	Organic acids	
X15	5.58	C_7_H_6_O_2_	4.3	121.0295	121.0302	50808	92.0266, 77.0403	*p*-Hydroxybenzaldehyde	Phenols	[14]
X16	5.64	C_9_H_8_O_4_	0.9	179.0350	179.0352	80360	134.0371, 108.0214, 93.0345, 89.0401, 79.0555	Caffeic acid	Organic acids	[13]
X17	5.99	C_8_H_8_O_4_	3	167.0350	167.0355	275549	123.0454, 79.0570	Vanillic acid	Organic acids	[12]
X18^b^	7.31	C_19_H_28_O_12_	0.4	447.1508	447.1510	14270	269.1028, 233.0656, 161.0449, 101.0242, 71.0153	Gastrodin A	Glycosides	[12]
X19	7.68	C_17_H_19_N_5_O_5_	−1.6	372.1313	372.1307	2202	336.1054, 266.0867, 134.0467	*p*-Hydroxybenzyl adenosine	Nitrogen-containing compounds	
X20	7.73	C_8_H_8_O_3_	0.5	151.0401	151.0402	7660	136.0155, 108.0219, 92.0273	Vanilline	Phenols	
X21	8.62	C_17_H_20_O_9_	−0.7	367.1035	367.1032	21071	193.0496, 191.0553, 173.0455, 134.0369, 111.0463, 93.0349, 85.0299	3-Feruloylquinic acid	Organic acids	[16]
X22	8.94	C_9_H_10_O_3_	−0.8	165.0557	165.0556	2085	119.0515, 103.0604	L-(-)-Phenyllactic acid	Organic acids	
X23^b^	9.11	C_32_H_40_O_19_	4.9	727.2091	727.2151	122722	459.1156, 441.1045, 423.0937, 397.1142, 369.1188, 217.0496	Parishin B	Glycosides	[13]
X24	9.78	C_33_H_42_O_20_	4	757.2197	757.2227	659	503.1631, 453.0961, 161.0480	Parishin H or M	Glycosides	[12]
X25	10.00	C_10_H_10_O_4_	0.7	193.0506	193.0508	97733	178.0265, 149.0591, 134.0372	Ferulic acid	Organic acids	[13]
X26	10.02	C_21_H_28_O_13_	0	487.1457	487.1457	4684	441.0958, 423.0934, 397.1169, 161.0430, 111.0091	Parishin O or N	Glycosides	[12]
X27	10.05	C_32_H_40_O_19_	4.9	727.2091	727.2127	122722	459.1156, 441.1045, 423.0937, 397.1142, 369.1188, 161.0453	Parishin C	Glycosides	[12]
X28	10.71	C_14_H_14_O_3_S	−2.2	261.0591	261.0585	777	205.8269, 167.8694, 137.0057	4,4′-Dihydroxybenzyl sulfoxide	Phenols	[14]
X29	11.74	C_11_H_12_O_5_	−2.2	223.0612	223.0607	2431	108.0226, 179.0713	Sinapic acid	Organic acids	[13]
X30	12.20	C_7_H_6_O_3_	2.5	137.0244	137.0248	31464	108.0215, 93.0352, 65.0410	*p*-Hydroxybenzoic acid	Phenols	[14]
X31	13.21	C_45_H_56_O_25_	4.7	995.3038	995.3107	39727	727.2129, 441.1065, 423.0915, 397.1119, 161.0457	Parishin	Glycosides	[12]
X32	13.56	C_20_H_24_O_8_	0.2	391.1398	391.1399	6858	229.0860, 123.0452, 121.0288, 107.0511, 93.0357, 71.0265	Bis-(4-hydroxybenzyl)-ether-mono-β-D-glucopyranoside	Glycosides	[14]
X33	13.69	C_26_H_30_O_14_	2.9	565.1563	565.1579	811	177.0188, 111.0153	Parishin W	Glycosides	[12]
X34	14.13	C_25_H_24_O_12_	2.6	515.1195	515.1208	91406	353.0879, 335.0781, 191.0558, 179.0345, 173.0450, 161.0240	3,4-Dicaffeoylquinic acid isomer	Organic acids	[15]
X35	16.48	C_25_H_24_O_12_	2.6	515.1195	515.1208	91406	353.0879, 335.0781, 191.0558, 179.0345, 173.0450, 161.0240	3,4-Dicaffeoylquinic acid isomer	Organic acids	[15]
X36	17.42	C_9_H_12_O_2_	−0.8	151.0765	151.0763	1891	133.0677, 107.0520, 94.0274, 77.0394	4-(Ethoxymethyl)phenol	Phenols	[14]
X37	23.89	C_14_H_14_O_2_S	−4	245.0642	245.0632	2498	139.0217	Bis(4-hydroxybenzyl)sulfide	Phenols	[14]
X38	32.90	C_9_H_8_O_3_	−1	163.0401	163.0399	4130	145.0279, 135.0455, 119.0493, 91.0184, 77.0443, 65.0039	*p*-Hydroxycinnamic acid	Organic acids	
X39	39.73	C_16_H_22_O_4_	−0.2	277.1445	277.1445	5380	233.1544, 206.8262	Dibutyl phthalate	Phenols	
Peak	RT (min)	Formula	Error (ppm)	[M+H]^+^ (*m*/*z*)		Intensity	Product ions	Identification	Structure class	Ref.
				Calculated	Measure					
Y1^a,b^	1.05	C_5_H_5_N_5_	−1.8	136.0618	136.0615	46698	nd	Adenine	Nitrogen-containing compounds	
Y2	1.08	C_10_H_13_N_5_O_4_	1.5	268.1040	268.1044	174357	136.0623, 119.0360,	Adenosine	Nitrogen-containing compounds	
Y3^b^	1.09	C_9_H_11_NO_3_	−1.9	182.0812	182.0808	34675	nd	Tyrosin	Nitrogen-containing compounds	
Y4	8.60	C_24_H_29_N_3_O_8_S	−0.5	520.1748	520.1746	7896	308.0836, 285.0913, 233.0591, 179.0486, 162.0208, 107.0485	(2)-g-L-[N-(4-Hydroxy benzyl)]glutamyl-L-[s-(4-hydroxybenzyl)] cysteinylglycine	Nitrogen-containing compounds	
Y5	9.77	C_10_H_8_O_4_	−0.8	193.0495	193.0494	14081	178.0257, 150.0323, 133.0281, 122.0361	3-Carboxyethyl-phthalide	Organic acids	[16]
Y6	10.55	C_12_H_18_O_5_	−0.1	243.1227	243.1227	13011	165.0909, 151.0381, 137.0949	3-Butyl-3-hydroxy-4,5,6,7-tetrahydro-6,7-dihydroxy phthalide	Organic acids	
Y7	10.55	C_12_H_16_O_4_	−0.5	225.1121	225.1119	21174	207.1023, 165.0557, 151.0376, 137.0954	Senkyunolide H or I or ligustilidiol or *cis*-6,7-dihydroxy-ligustilide	Phthalides	[9]
Y8	11.94	C_12_H_16_O_5_	−0.8	241.1071	241.1069	1918	150.0677, 107.0497, 71.0498	Senkyunolide R or S	Phthalides	
Y9	13.27	C_12_H_16_O_3_	0.1	209.1172	209.1172	288915	153.0544, 149.0593, 135.0473, 121.1006, 117.0709	Senkyunolide G or K or Z-6,7-epoxyligustilide	Phthalides	[9]
Y10	13.27	C_12_H_18_O_4_	0.4	227.1278	227.1279	102387	163.1104, 153.0543, 149.0961, 119.0860, 107.0484, 79.054	Senkyunolide J or N or R2	Phthalides	[9]
Y11	16.56	C_12_H_14_O_3_	−0.6	207.1016	207.1015	1327567	189.0917, 165.0550, 146.0732, 133.0653, 119.0841, 105.0693	3-Butylidene-6-hydroxy-5,6-dihy-drophthalide or senkyunolide F or chuanxiongol	Phthalides	[9]
Y12	16.57	C_12_H_16_O_4_	−0.9	225.1121	225.1119	39845	165.0533, 133.0658, 128.0619, 91.0532, 77.0402	Senkyunolide H or I or ligustilidiol or *cis*-6,7-dihydroxy-ligustilide	Phthalides	[9]
Y13	17.87	C_12_H_14_O_4_	−0.2	223.0965	223.0966	23855	177.0921, 149.0591, 121.0308, 103.0523, 77.0401	Senkyunolide D or 4,7-dihydroxy-3-butylphthalide	Phthalides	[9]
Y14	18.08	C_12_H_14_O_3_	−0.1	207.1016	207.1015	425060	189.0901, 165.0558, 161.0948, 128.0620, 105.0701, 91.0549, 77.0393	3-Butylidene-6-hydroxy-5,6-dihy-drophthalide or senkyunolide F or chuanxiongol	Phthalides	[9]
Y15	19.53	C_18_H_28_O_8_	0.3	373.1857	373.1858	45450	211.1335, 193.1228, 147.1172, 105.0702, 79.0577	Ligusticoside A	Glycosides	[17]
Y16	21.97	C_12_H_12_O_2_	−0.5	189.0910	189.0910	151946	128.0623, 115.0544, 105.0702, 91.0551, 77.0388	Butylidenephthalide isomer	Phthalides	[9]
Y17	23.67	C_12_H_14_O_4_	0.3	223.0965	223.0966	43428	177.0899, 167.0387, 149.0227, 121.0278, 91.0541, 77.0382	Senkyunolide D or 4,7-dihydroxy-3-butylphthalide	Phthalides	[9]
Y18	24.29	C_12_H_12_O_2_	0	189.0910	189.0910	305525	152.0611, 128.0618, 115.0529, 105.0341, 91.0537, 77.0394	Butylidenephthalide isomer	Phthalides	[9]
Y19	24.50	C_12_H_14_O_2_	−0.9	191.1067	191.1067	340079	117.0688, 91.0560, 77.0396	3-Butylphthalide or Z-ligustilide or E-ligustilide	Phthalides	[9]
Y20	26.27	C_12_H_15_ClO_3_	−0.5	243.0783	243.0781	21244	207.1020, 189.0907, 161.0364, 133.0640, 119.0840, 91.0533,	Senkyunolide L	Phthalides	[18]
Y21	26.63	C_12_H_12_O_3_	0.6	205.0859	205.0860	90627	187.0745, 168.0574, 144.0573, 131.0493, 115.0541, 103.0552, 91.0533, 77.0401	Senkyunolide B or C	Phthalides	[9]
Y22	27.74	C_13_H_16_O_3_	−0.6	221.1172	221.1171	4804	175.1090, 105.0691	2-(1-Oxopentyl)-benzoic	Organic acids	
Y23	27.90	C_12_H_12_O_3_	0.6	205.0859	205.0860	90627	187.0745, 168.0574, 144.0573, 131.0493, 115.0541, 103.0552, 91.0533, 77.0401	Senkyunolide B or C	Phthalides	[9]
Y24	28.65	C_12_H_16_O_2_	0.7	193.1223	193.1225	2273337	147.1167, 137.0590, 119.0498, 105.0699, 91.0544, 77.0389, 65.0383	Senkyunolide A	Phthalides	[9]
Y25	29.49	C_12_H_14_O_2_	0.3	191.1067	191.1067	3489472	153.0704, 145.1007, 135.0440, 117.0695, 91.0546, 77.0391	3-Butylphthalide or Z-ligustilide or E-ligustilide	Phthalides	[9]
Y26	32.06	C_12_H_12_O_2_	−0.5	189.0910	189.0910	39396	152.0611, 128.0615, 115.0545, 91.0554	Butylidenephthalide isomer	Phthalides	[9]
Y27^b^	32.89	C_12_H_18_O_2_	−0.8	195.1380	195.1378	686098	177.1344, 149.1309, 79.0550	Cnidilide or neocnilide	Phthalides	[9]
Y28	33.04	C_12_H_14_O_2_	0.3	191.1067	191.1067	637725	173.0961, 145.1008, 130.0773, 117.0694, 91.0548, 77.0392	3-Butylphthalide or Z-ligustilide or E-ligustilide	Phthalides	[9]
Y29	33.64	C_12_H_12_O_2_	−0.2	189.0910	189.0910	401466	152.0627, 128.0622, 115.0540, 91.0550	Butylidenephthalide isomer	Phthalides	[9]
Y30	35.24	C_12_H_20_O_2_	−0.7	197.1536	197.1535	21102	119.0862, 95.0856, 91.0555, 81.0697, 67.0552,	3,7-Dimethyl-3-acetate-1,6-octadiene-3-ol acetate	Others	
Y31	36.15	C_21_H_32_O_2_	0.9	317.2475	317.2478	12477	281.2253, 211.1524, 187.1501, 159.1179, 149.1329, 145.0997, 117.0717, 81.0720	Pregnenolone	Others	
Y32	36.37	C_24_H_28_O_5_	0.1	397.2010	397.2010	71592	191.1064, 173.0954, 155.0852, 145.1003, 128.0625, 117.0715, 105.0724, 91.0544	Chuanxiongnolide A or B	Others	[19]
Y33	38.22	C_24_H_28_O_5_	0.4	397.2010	397.2010	66600	361.1802, 333.1842, 307.1708, 291.1401, 279.1403, 261.1264,2 17.0999, 191.1070, 173.0972, 91.0553	Chuanxiongnolide A or B	Others	[19]
Y34	39.08	C_24_H_32_O_5_	−0.4	401.2323	401.2321	20533	383.2246, 191.1061, 163.1084, 149.0594, 145.1037, 135.0439	Chuanxiongdiolide R2 or chuanxiongdiolide B	Others	[20]
Y35	39.48	C_17_H_24_O_4_	0.8	293.1747	293.1750	22471	175.1115, 151.0754, 137.0605, 111.0415, 91.0547, 83.0488	Senkyunolide M or Q	Phthalides	[9]
Y36	41.42	C_24_H_30_O_4_	−0.1	383.2217	383.2217	1160225	191.1068, 173.0958, 163.1120, 149.0601, 135.0440, 91.0546, 79.0566	Senkyunolide P or 3,8-dihydro-diligustilide or angelicide or Z,Z′−3,3′ −8,8′-diligustilide	Phthalides	[9]
Y37	41.52	C_24_H_32_O_4_	0.1	385.2373	385.2374	627373	367.2247, 349.2092, 321.2178, 293.1915, 193.1229, 175.1113147.1159, 137.0587, 119.0871, 93.0687	Chuanxiongdiolide A	Others	[20]
Y38	41.88	C_24_H_28_O_4_	0.1	381.2060	381.2061	4796897	191.1070, 173.0955, 163.1126, 149.0596, 135.0437, 91.0551, 79.0549	Levistolide A or senkyunolide O or tokinolide B or riligustilide	Phthalides	[9]

^a^“X” in negative ion mode and “Y” in negative-positive mode. ^b^Compared with reference standards.

**Table 2:** Metabolites identified in plasma of rats after oral administration by UPLC/Q-TOF MS.

No.	Parent compounds	Metabolic pathways	Formula	tR (min)	[M+H]^+^ (*m*/*z*)		Error (ppm)	Product ions
					Calculated	Measure		
1	4,7-Dihydroxy-3-butylphthalide, senkyunolide D	Prototype	C_12_H_14_O_4_	9.75	223.0965	223.0963	−1	177.0899, 167.0387, 149.0226, 121.0278, 91.0541
2	4,7-Dihydroxy-3-butylphthalide, senkyunolide D	Hydroxyl and methyl conjugation	C_13_H_16_O_5_	10.78	253.1071	253.1071	0.2	235.0963, 221.0829, 202.0596, 193.0489, 179.0332, 175.0379, 150.0301, 121.0268
3	4,7-Dihydroxy-3-butylphthalide, senkyunolide D	Sulfate conjugation	C_12_H_14S_O_7_	9.2	303.0533	303.0535	0.5	285.0430, 205.0858, 187.0753, 177.0904, 149.0244, 121.0283, 91.0534
4	4,7-Dihydroxy-3-butylphthalide, senkyunolide D	Glucuronide conjugation	C_18_H_22_O_10_	10.6	399.1286	399.1285	−0.3	223.0969, 205.0847, 177.0883, 167.0331, 149.0233, 121.0315
5	4,7-Dihydroxy-3-butylphthalide, senkyunolide D	Hydrogenation and glucuronide conjugation	C_18_H_24_O_10_	8.67	401.1442	401.1444	0.3	225.4427, 207.1017, 189.0924, 172.0884, 165.0548, 141.0170, 119.0851, 113.0288
6	4,7-Dihydroxy-3-butylphthalide, senkyunolide D	Hydroxyl and glucuronide conjugation	C_18_H_22_O_11_	8.92	415.1235	415.1238	0.7	227.0584, 221.0824, 167.0331
7	3-Butyl-3-hydroxy-4,5,6,7-tetrahy-dro-6,7-dihydroxyphthalide	Prototype	C_12_H_18_O_5_	7.76	243.1227	243.1225	−0.7	165.0909, 151.0414, 137.0951, 123.0431, 107.0499, 91.0546, 85.0648
8	3-Butyl-3-hydroxy-4,5,6,7-tetrahy-dro-6,7-dihydroxyphthalide	Methyl conjugation	C_13_H_20_O_5_	10.01	257.1384	257.1381	−1	221.1211, 207.0993, 165.0913, 137.0951, 123.0434
9	3-Butyl-3-hydroxy-4,5,6,7-tetrahy-dro-6,7-dihydroxyphthalide	Cystein conjugation	C_15_H_23_O_6_NS	7.67	346.1319	346.1319	0	328.1222, 310.1111, 264.1056, 238.0916, 223.0771, 207.1018, 165.0923, 137.0955
10	3-Butyl-3-hydroxy-4,5,6,7-tetrahy-dro-6,7-dihydroxyphthalide	Oxidation and cystein conjugation	C_15_H_23_O_7_NS	9.47	362.1268	362.1267	−0.3	327.0911, 247.1337, 229.1216, 151.0746
11	3-Butyl-3-hydroxy-4,5,6,7-tetrahy-dro-6,7-dihydroxyphthalide	*N*-Acetyl-L-cysteine conjugation	C_17_H_25_O_8_NS	5.97	404.1374	404.1372	−0.5	205.0833, 171.1364
12	3-Butyl-3-hydroxy-4,5,6,7-tetrahy-dro-6,7-dihydroxyphthalide	Desat and S-GSH conjugation	C_22_H_33_O_11_N_3S_	4.39	548.1909	548.1921	2.3	473.1636, 419.1465, 205.0860
13	3-Butyl-3-hydroxy-4,5,6,7-tetrahy-dro-6,7-dihydroxyphthalide	H_2_O conjugation	C_12_H_20_O_6_	4.65	261.1333	261.1320	−4.6	261.1310
14	3-Carboxyethyl-phthalide	Prototype	C_10_H_8_O_4_	7.28	193.0495	193.0490	−2.7	178.0257, 150.0323, 133.0277, 122.0361, 105.0338, 77.0388
15	3-Carboxyethyl-phthalide	Methyl conjugation	C_11_H_10_O_4_	11.52	207.0652	207.0646	−3	147.0441, 131.0502, 103.0546, 91.0533
16	3-Carboxyethyl-phthalide	Glucuronide conjugation	C_16_H_16_O_10_	5.51	369.0816	369.0822	1.5	193.0493
17	3-Carboxyethyl-phthalide	Hydroxyl and glucuronide conjugation	C_16_H_16_O_11_	5.72	385.0765	385.0758	−2.1	209.0455
18	3-Carboxyethyl-phthalide	Hydrogenation	C_10_H_10_O_4_	7.64	195.0652	195.0651	−0.6	177.0547, 149.0609, 145.0276, 134.0354, 117.0309, 89.0395
19	Cnidilide, neocnilide	Prototype	C_12_H_18_O_2_	16.14	195.1380	195.1377	−1.5	177.1350, 149.1348, 125.0599, 107.0873, 97.0640, 91.0550, 79.0543
20	Cnidilide, neocnilide	Methyl conjugation	C_13_H_20_O_2_	15.63	209.1536	209.1537	0.4	193.0211, 167.1088, 153.0917, 121.0648, 68.9961
21	Cnidilide, neocnilide	Acetyl conjugation	C_14_H_20_O_3_	12.45	237.1485	237.1483	−1.1	177.0257
22	Cnidilide, neocnilide	Hydroxyl and acetyl conjugation	C_14_H_20_O_4_	12.46	253.1434	253.1435	0.4	235.1340, 193.0856, 157.1012, 135.0816
23	Cnidilide, neocnilide	Oxidation and cystein conjugation	C_15_H_23_O_4_NS	10.07	314.1421	314.1424	1.1	268.1343, 193.1185
24	Cnidilide, neocnilide	Hydroxyl and glucuronide conjugation	C_18_H_26_O_9_	9.62	387.1650	387.1650	0.1	211.1330, 193.1223, 175.1129, 147.1168, 121.0368
25	Cnidilide, neocnilide	2Hydroxyl and glucuronide conjugation	C_18_H_26_O_10_	8.02	403.1599	403.1598	−0.2	227.1287, 209.1174, 191.1065, 171.1373, 163.1123, 153.0549, 145.1025, 141.0186, 135.1164, 121.0995
26	Cnidilide, neocnilide	2Hydroxyl conjugation	C_12_H_18_O_4_	8.68	227.1278	227.1279	0.7	191.1057, 163.1115, 153.0554, 145.1001, 135.0444, 105.0705, 91.0541
27	Senkyunolide A	Prototype	C_12_H_16_O_2_	14.81	193.1223	193.1222	−0.5	175.1118, 147.1170, 137.0580, 119.0848, 105.0710, 91.0556, 77.0393
28	Senkyunolide A	Hydroxyl and methyl conjugation	C_13_H_18_O_3_	15.85	223.1329	223.1326	−1.2	205.1224, 191.1060, 149.0235, 135.0429, 121.0279, 105.0697, 91.0542, 77.0397
29	Senkyunolide A	Hydroxyl and acetylcysteine conjugation	C_17_H_25_O_6_NS	10.35	372.1475	372.1475	−0.1	330.1375, 284.1322, 267.1048.239.0756, 209.1169, 191.1074, 162.0210, 153.0540, 130.0492
30	Senkyunolide A	Carboxyl and glucuronide conjugation	C_18_H_22_O_10_	10.6	399.1286	399.1285	−0.3	223.0969, 205.0847, 177.0883, 159.0293, 149.0233, 131.0840, 85.0275
31	Senkyunolide A	2Hydroxyl and glucuronide conjugation	C_18_H_24_O_10_	8.67	401.1442	401.1444	0.3	225.1127, 207.1017, 189.0924, 172.0884, 165.0548, 141.0170, 119.0851, 113.0288, 85.0265, 73.0295
32	Senkyunolide A	H_2_O conjugation	C_12_H_18_O_3_	10.54	211.1329	211.1327	−0.9	193.1225, 175.1096, 147.1156, 129.0700, 105.0693, 93.0692, 79.0548
33	Senkyunolide A	2Hydroxyl conjugation	C_12_H_16_O_4_	9.56	225.1121	225.1119	−1	207.1013, 189.0914, 165.0537, 133.0637, 105.0706, 91.0536, 81.0713
34	Senkyunolide G, senkyunolide K,Z-6,7-epoxyligustilide	Prototype	C_12_H_16_O_3_	12.79	209.1172	209.1173	0.3	153.0686, 149.0594, 145.0984, 135.0472, 105.0693, 91.0562, 77.0409
35	Senkyunolide G, senkyunolide K,Z-6,7-epoxyligustilide	Methyl conjugation	C_13_H_18_O_3_	15.85	223.1329	223.1326	−1.2	191.1060, 173.0946, 149.0235, 145.1014, 135.0429, 105.0697, 91.0542, 79.0551
36	Senkyunolide G, senkyunolide K,Z-6,7-epoxyligustilide	Acetyl conjugation	C_14_H_18_O_4_	15.99	251.1278	251.1276	−0.6	177.1261, 149.0593, 69.0014, 57.0752
37	Senkyunolide G, senkyunolide K,Z-6,7-epoxyligustilide	Hydroxyl and acetyl conjugation	C_14_H_18_O_5_	12.27	267.1227	267.1226	−0.4	249.1137, 193.0479, 189.0582, 135.0435, 119.0846
38	Senkyunolide G, senkyunolide K,Z-6,7-epoxyligustilide	Taurine conjugation	C_15_H_21_O_5_NS	9.02	328.1213	328.1214	0.1	207.1015, 189.0911, 165.0541, 161.0955, 147.0814, 133.0644, 119.0859, 91.0538
39	Senkyunolide G, senkyunolide K,Z-6,7-epoxyligustilide	*N*-Acetyl-L-cysteine conjugation	C_17_H_23_O_6_NS	11.15	370.1319	370.1320	0.3	282.1162, 264.1083, 207.1015, 189.0911, 165.0544, 147.0785, 133.0652, 119.0858, 91.0546
40	Senkyunolide G, senkyunolide K,Z-6,7-epoxyligustilide	Hydrogenation and glucuronide conjugation	C_18_H_26_O_9_	9.62	387.1650	387.1650	0.1	211.1330, 193.1223, 175.1129, 147.1168, 121.0638, 91.0546, 79.0562
41	Senkyunolide G, senkyunolide K,Z-6,7-epoxyligustilide	Hydroxyl and acetylcysteine conjugation	C_17_H_25_O_7_NS	8.29	388.1425	388.1422	−0.6	207.1010, 164.0390, 122.0273, 105.0347, 79.0529
42	Senkyunolide G, senkyunolide K,Z-6,7-epoxyligustilide	Hydroxyl and glucuronide conjugation	C_18_H_24_O_10_	8.67	401.1442	401.1444	0.3	267.1015, 225.1127, 207.1017, 189.0924, 172.0844, 141.0170, 113.0228, 85.0265, 73.0295
43	Senkyunolide G, senkyunolide K,Z-6,7-epoxyligustilide	Carboxyl and glucuronide conjugation	C_18_H_22_O_11_	8.92	415.1235	415.1238	0.7	227.0584, 221.0824
44	Senkyunolide G, senkyunolide K,Z-6,7-epoxyligustilide	Desat and S-GSH conjugation	C_22_H_31_O_9_N_3_S	9.17	514.1854	514.1854	0	439.1552, 385.1429, 282.1160, 207.1017, 189.0921, 179.0484, 162.0221, 144.0103, 116.0174, 76.0218
45	Senkyunolide G, senkyunolide K,Z-6,7-epoxyligustilide	S-GSH conjugation	C_22_H_33_O_9_N_3_S	8.61	516.2010	516.2010	−0.1	441.1694, 387.1593, 284.1315, 209.1173, 191.1055, 162.0212, 144.0109, 116.0175, 84.0447
46	Senkyunolide G, senkyunolide K,Z-6,7-epoxyligustilide	Hydrogenation	C_12_H_18_O_3_	10.54	211.1329	211.1327	−0.9	193.1225, 175.1096, 151.0737, 147.1156, 129.0700, 121.0641, 105.0693, 93.0693, 91.0546, 77.0398
47	Senkyunolide G, senkyunolide K,Z-6,7-epoxyligustilide	Hydroxyl and demethyl conjugation	C_12_H_14_O_4_	9.75	223.0965	223.0963	−1	177.0906, 149.0590, 145.0980, 105.0329, 91.0556, 77.0394
48	Senkyunolide G, senkyunolide K,Z-6,7-epoxyligustilide	H_2_O conjugation	C_12_H_18_O_4_	8.68	227.1278	227.1279	0.7	209.1169, 191.1057, 163.1115, 153.0554, 145.1001, 135.0444, 105.0705, 91.0541, 77.0388, 65.0402, 55.0198
49	Senkyunolide G, senkyunolide K,Z-6,7-epoxyligustilide	Demethyl and carboxyl conjugation	C_12_H_14_O_5_	9.79	239.0914	239.0915	0.4	221.0816, 193.0885, 179.0336, 165.0173, 161.0227, 128.0633, 109.0292, 77.0376
50	Senkyunolide G, senkyunolide K,Z-6,7-epoxyligustilide	2hydrogenation and 2hydroxyl conjugation	C_12_H_18_O_5_	7.76	243.1227	243.1225	−0.7	225.1135, 207.1017, 179.1084, 165.0909, 151.0414, 137.0951, 123.0431, 95.0486
51	Senkyunolide G, senkyunolide K,Z-6,7-epoxyligustilide	Aromatic hydrocarbon oxidation	C_13_H_18_O_5_	11.38	255.1227	255.1222	−2.1	195.1024, 135.0798, 131.0870
52	Senkyunolide J,N,R2	Prototype	C_12_H_18_O_4_	8.68	227.1278	227.1279	0.7	163.1115, 153.0554, 145.1001, 107.0705, 91.0541, 79.0544, 65.0402, 55.0198
53	Senkyunolide J,N,R2	Methyl conjugation	C_13_H_20_O_4_	11.42	241.1434	241.1433	−0.6	209.1147, 191.1067, 163.1089, 153.0559, 121.0652, 93.0717, 85.0663, 79.0663, 57.0698
54	Senkyunolide J,N,R2	Hydroxyl and methyl conjugation	C_13_H_20_O_5_	10.01	257.1384	257.1381	−1	221.1211, 207.0993, 165.0913, 161.0984, 137.0951, 123.0434, 93.0699, 79.0549, 67.0538
55	Senkyunolide J,N,R2	Glycine conjugation	C_14_H_21_O_5_N	7.49	284.1493	284.1495	0.8	238.1464, 209.1195, 191.1065, 163.1151, 153.0546, 135.1157, 117.0704, 91.0562, 76.0410, 57.0711
56	Senkyunolide J,N,R2	Cystein conjugation	C_15_H_23_O_5_NS	8.48	330.1370	330.1375	1.7	284.1324, 209.1176, 191.1066, 163.1120, 153.0544, 145.1015, 121.1008, 93.0695, 85.0650
57	Senkyunolide J,N,R2	Taurine conjugation	C_14_H_23_O_6_NS	7.56	334.1319	334.1319	0	209.1147, 181.1211, 153.0555, 126.0211, 108.0102, 91.0549
58	Senkyunolide J,N,R2	Oxidation and cystein conjugation	C_15_H_23_O_6_NS	7.67	346.1319	346.1319	0	328.1222, 310.1111, 264.1056, 238.0916, 207.1018, 195.0853, 165.0923, 137.0955
59	Senkyunolide J,N,R2	*N*-Acetyl-L-cysteine conjugation	C_17_H_25_O_7_NS	8.29	388.1425	388.1422	−0.6	207.1010, 164.0390, 107.0491, 122.0273, 105.0347
60	Senkyunolide J,N,R2	Glucuronide conjugation	C_18_H_26_O_10_	8.02	403.1599	403.1598	−0.2	227.1287, 209.1174, 191.1065, 163.1123, 153.0549, 145.1025, 135.1164, 121.0995, 93.0712
61	Senkyunolide J,N,R2	S-GSH conjugation	C_22_H_35_O_10_N_3_S	4.96	534.2116	534.2113	−0.6	459.1795, 405.1696, 387.1582, 369.1490, 341.1469, 302.1438, 284.1303, 284.1303, 241.0913, 209.1157, 191.1075

## Data Availability

The data used to support the findings of this study are available from the corresponding author upon request.
